# Assessing Changes in Dielectric Properties Due to Nanomaterials Using a Two-Port Microwave System

**DOI:** 10.3390/s20216228

**Published:** 2020-10-31

**Authors:** Mohammed Rahman, Rachita Lahri, Syed Ahsan, Maya Thanou, Panagiotis Kosmas

**Affiliations:** 1 Institute of Pharmaceutical Sciences, King’s College London, Strand, London WC2R 2LS, UK; mohammed.3.rahman@kcl.ac.uk (M.R.); rachita3.lahri@gmail.com (R.L.); maya.thanou@kcl.ac.uk (M.T.); 2 Faculty of Natural and Mathematical Sciences, King’s College London, Strand, London WC2R 2LS, UK; sayyed.n.ahsan@gmail.com

**Keywords:** microwave imaging, nanoparticles, contrast enhancement

## Abstract

Detecting changes in the dielectric properties of tissues at microwave frequencies can offer simple and cost effective tools for cancer detection. These changes can be enhanced by the use of nanoparticles (NPs) that are characterised by both increased tumour uptake and high dielectric constant. This paper presents a two-port experimental setup to assess the impact of contrast enhancement on microwave signals. The study focuses on carbon nanotubes, as they have been previously shown to induce high microwave dielectric contrast. We investigate multiwall carbon nanotubes (MWNT) and their -OH functionalised version (MWNT-OH) dispersed in tissue phantoms as contrast enhancing NPs, as well as salt (NaCl) solutions as reference mixtures which can be easily dissolved inside water mixtures and thus induce dielectric contrast changes reliably. MWNT and MWNT-OH are characterised by atomic force microscopy, and their dielectric properties are measured when dispersed in 60% glycerol–water mixtures. Salt concentrations between 10 and 50 mg/mL in 60% glycerol mixtures are also studied as homogeneous samples known to affect the dielectric constant. Contrast enhancement is then evaluated using a simplified two-port microwave system to identify the impact on microwave signals with respect to dielectric contrast. Numerical simulations are also conducted to compare results with the experimental findings. Our results suggest that this approach can be used as a reliable method to screen and assess contrast enhancing materials with regards to a microwave system’s ability to detect their impact on a target.

## 1. Introduction

Microwave tomography (MWT) and imaging (MWI) are currently being developed for various applications [1,2,3]. In breast cancer detection, for example, MWI is currently tested using both qualitative (radar-based) and quantitative (tomography) techniques. MWI systems utilise non-ionising, low power microwaves, and have the potential to be inexpensive, portable, and produce images in real (or quasi-real) time. Clinical studies such as those of the Dartmouth MWT system [3] and the MARIA M4 radar-based imaging system [4] indicate that MWI has potential to provide a reliable and safe alternative tumor detection method.

Various dielectric spectroscopy studies [5,6,7,8,9] of excised healthy and cancerous tissues have suggested that the dielectric contrast between (dense) healthy and cancerous breast tissue can be as low as 10%. These tissue characterisation studies show variation in dielectric measurements, which can be explained by the sample preparation and measurement technique. The accuracy of dielectric spectroscopy [10,11] and the impact of the tissue sample’s hydration level [12,13] complicate further the goal of assessing tissue contrast based on dielectric spectroscopy measurements. Indeed, tissue hydration can pose challenges in producing consistent dielectric measurements, as it can affect the complex dielectric constant. For example, dielectric measurements of excised rodent tissues (fat and muscle) were recently compared with in vivo measurements and showed a correlation between dehydration and reduced dielectric properties [14]. Dielectric profiles of excised rodent liver tissues were taken over time in [15], which showed a similar trend of dielectric properties being reduced because of dehydration. Dielectric characterisations of breast cancer cells have also been conducted to further assess the dielectric components in the tumours [16,17].

Contrast agents are used widely in imaging and screening techniques to overcome issues of poor low contrast between healthy and malignant tissue [18]. Contrast-enhanced MW imaging has been studied in clinical scenarios, but mostly with hypothetical simulation models [19,20]. Clinically accepted contrast agents must be biocompatible and chemically modified for intravenous delivery. Their size should be suitable to take advantage of the enhanced permeation and retention effect observed in solid tumours, and they should induce detectable dielectric contrast for radar and tomographic MWI techniques. Moreover, contrast agents should show preferential accumulation to the tumours and achieve high contrast concentration in the tumour region. To this end, the contrast agents can be decorated with specific antibodies for cancer biomarkers, such as trastuzumab antibodies in the case of breast cancer [21,22].

Various studies have looked at the possibility of microwave contrast enhancement for biocompatible agents such as functionalised iron oxide NPs [23]. Dispersed iron oxide NPs have shown magnetic contrast which can be detected in phantoms using a microwave setup coupled with a magnet to turn on and off the polarizing magnetic field [24,25,26]. Recent work on metal-based nanomaterials suggests that ferrite and titanate NPs may have a strong effect on the dielectric properties of tissues. These NPs are characterised by a more biocompatible profile relative to carbon nanotubes [27]. Gold nanoparticles have also been proposed as a promising biocompatible alternative to other metal contrast agents, but previous studies have shown very limited ability on affecting the dielectric constant of water [28,29].

Our group has identified highly dispersible and biocompatible zinc ferrite NPs as agents that may induce detectable dielectric contrast in a background medium at microwave frequencies. These particles may be also able to increase the conductivity of the targeted region, suggesting potential to be used as thermal agents for localised microwave hyperthermia [30]. PEGylated zinc oxide NPs have also been shown to induce dielectric contrast in aqueous suspensions, and their resulting dielectric properties are heavily dependent on the molecular weight of the polyethylene glycol [31,32]. Reduced graphene oxide (RGO) and modified RGO with zinc oxide nanowires (ZnO/RGO) have also shown high dielectric properties at higher frequencies, between 8.0 and 12.0 GHz [33,34]. Prior to these studies, carbon nanotubes (CNTs) were proposed as nanomaterials that can induce microwave dielectric contrast. Standard CNTs were shown to exhibit dielectric contrast over ∼40% and conductivity contrast of ∼80% [35], and experiments of dispersing CNTs into breast tumour mimicking phantoms have been reported [36]. Moreover, it has been shown that purification method and solvent choice have significant affect on the CNTs dielectric properties [37,38].

CNTs have unique electrical properties, which can be attributed to their distinctive structure [39]. As such, they have been characterised in the microwave spectrum to identify their benefits for several applications. They have also been assessed as potential microwave nano-absorbers and have been modified in several ways to enhance these properties [40]. For example, single-walled carbon nanotubes (SWCNTs) filled with cobalt nanoparticles and dispersed in cured epoxy resin has shown enormous dielectric and magnetic contrast with respect to the controls between 2.0 and 18.0 GHz, as measured by the coaxial transmission line technique [41]. Similarly, multi-walled carbon nanotubes (MWNTs) filled with samarium oxide nanoparticles have shown significant dielectric contrast in the order of ∼40% compared with pristine MWNTs in the range of 2.0–14.0 GHz [42]. The microwave characterisation of Nickle-filled MWNTs dispersed in paraffin also demonstrated that doubling the concentration of the composite increases significantly the permittivity and permeability properties between 2.0 and 18.0 GHz [43]. Iron filled MWNTs dispersed in olefin at 20%wt ratio have shown extreme dielectric loss compared with basic MWNTs, between 8 and 18 GHz [44,45]. Iron oxide NPs attached to the outer surface of MWNTs exhibit varying microwave absorption properties, when mixed with/out poly(N-vinyl-2-pyrrolidone) dispersant [46]. Fusion between CNTs and other metallic and semiconductor materials offer higher microwave absorption capacities [47,48].

Based on the above, this work uses MWNTs as a nanomaterial reference for assessing the impact of contrast enhancement at microwave frequencies. In particular, our aim is to suggest a methodology which can assess whether the impact of dielectric contrast enhancement can be detected in a MWI system. Our results can serve as reference for future studies of more biocompatible but less strong contrast-enhancing agents such as the PEGylated zinc oxide NPs that we have proposed recently [31,32]. To this end, we have employed an experimental two-port microwave measurement system, basic and functionalised multi-walled carbon nanotubes (MWNT and MWNT-OH) as contrast agents, and varying concentrations of salt mixed in 60% glycerol as materials with the potential of altering the dielectric constant. Salt has two main functions in this study: first, to induce significant change in dielectric properties as observed in previous spectroscopy studies of saline [49] and aqueous-based materials [50]. Second, salt dissolves in aqueous solutions and is considered isotropic, thus presenting a reliable means of inducing consistent dielectric properties that depend on its salt concentration in the 60% glycerol water mixtures.

Our microwave experiments employ the same bi-static (two-port) microwave (2PMW) setup as in [51], but for a very different purpose. While experiments in [51] were set up to test reconstructions of high contrast dielectric targets, our focus here is to assess the impact of dielectric contrast enhancement on a potential MWI system for medical applications. In a clinical scenario, the contrast-enhanced target will represent a small region inside a lossy background medium, and the interaction of these two “regions” will have a significant impact on the measured signals. The lossy surrounding region will cause significant attenuation in the signal scattered by the contrast-targeted region, resulting in a very weak signal which may not be detectable by our measurement apparatus (VNA) for certain receiver positions (Rx) relative to the transmitting antenna (Tx). To answer these questions without over-complicating the measurement, we chose to use an imaging tank with a liquid phantom resembling fatty human tissue, a cylindrical target filled with a liquid phantom resembling malignant tissue with and without contrast agents, four Tx/Rx pairs, and antennas that can be realistic candidates for microwave medical imaging [51].

We note that our approach is more complicated and prone to errors than a direct measurement with two antennas facing the contrast-enhanced liquid phantom, which could produce a more reliable method to assess the impact of contrast enhancement on the microwave signals transmitted and received by the antenna pair. Our choice of a more complicated setup is motivated by our interest in studying how contrast enhancement can impact the signals produced and processed by a multi-view MWI system that we are currently developing. Our MWI system is intended to exploit signals that can be either mostly due to back-scatter from the target at oblique angles, or due to signals transmitted through the background medium and the target. The 2PMW setup with four Tx/Rx pairs studied in this paper serves this purpose as an initial investigation. Future work should extend this study to a fully multi-static system, as well as compare these findings to more robust measurements with an antenna pair only. This comparison would answer the question on how the impact of contrast enhancement measured by the antenna pair is translated to a system designed for a practical MWI application.

To evaluate the impact of contrast enhancement for our imaging prototype under development, we have selected a NP concentration of 2 mg/mL for the CNTs in agreement with previous studies [32,52]. We also note that carbon nanotubes are known to be toxic and non-biocompatible. Multiple pathways of administration have shown cytotoxic effects through in vitro and in vivo [53,54]. Recent in-vivo studies have shown success in making CNTs biocompatible for anti-cancer drugs [55,56] and bone regeneration [57]. However, these studies do not administer the NPs intravenously, and thus delivery to the targeted location without damaging other areas remains a challenge. Despite these limitations, the deployment of CNTs as contrast agents for our experiments is necessary as a reference for assessing the NP agent’s impact on microwave signals.

Our phantoms comprise only a background medium with a low dielectric constant (safflower oil) and a cylindrical target with a high dielectric constant (mixture of 60% glycerol and RO water), where the NPs or salt are introduced. Specifically, the dielectric constant for safflower oil is 2.9 at 1.0 GHz, which is comparable to the dielectric constant of fatty breast tissue in this frequency range [5,6,58]. The mixture of 60% glycerol and RO water has a high dielectric constant (56.6 at 1.0 GHz) and allows better dispersibility of the NPs. This high dielectric constant liquid can serve as initial phantom for tumour tissue, for which the dielectric constant varies between 50 and 60 [59,60,61]. To ensure that results are conclusive and not due to possible measurement errors, we have performed realistic simulations of our experiments which have reaffirmed our experimental findings.

## 2. Materials and Methods

### 2.1. Atomic Force Microscopy (AFM)

MWNT and MWNT-OH aqueous suspensions were prepared in water at 2 mg/mL, and 100 μL was pipetted on the glass microscopic slide which was dried using nitrogen gas. Images were taken using the Bruker icon dimension atomic force microscope (Bruker Corporation, Billerica, MA, USA), with the standard tapping mode applied. The scanning area started at a large region of 10.0 × 10.0 μm and was decreased to a smaller region of interest (ROI) of 1.7 × 1.7 μm. The scan rate decreased from 0.9 Hz to 0.7 Hz as the scan size decreased, to improve image quality. The software uses a PID feedback system to improve the trace and retrace signals. To improve the reliability of image acquisition, the scan angle of the probe is altered from 0${}^{\circ }$ to 90${}^{\circ }$ and the raster scan for the images is repeated. This is a confidence check to observe any artifacts produced because of particles stuck onto the cantilever.

### 2.2. Dielectric Characterisation

Dielectric characterisation was recorded using a an open-ended coaxial cable probe kit (85070E) (Keysight Technologies, Santa Rosa, CA, United States) and a performance vector analyser PNA E8362B (Keysight Technologies, Santa Rosa, California, United States). The slim-form probe was calibrated using three known dielectric materials: air, short block (conductive elastomer that mimics electrical properties of metal), and reverse osmosis purified (RO) water. The minimum and maximum frequencies for the dielectric measurements was set to 1.0–4.0 GHz, respectively. This frequency range is motivated by our aim to compare the impact of changes in dielectric contrast with the resulting changes in the microwave signals recorded by our experimental setup, which operates in the range of 1.0–4.0 GHz [51]. The probe operates in a much wider range, between 0.5 and 50 GHz, and needs a minimum of 5 mm sample thickness for measurements. We note that ideal measurements require that the probe is immersed into an isotropic medium. The complex permittivity for a given material is $\epsilon^{*}\left(\omega \right)=\epsilon^{\prime }\left(\omega \right)-j\epsilon^{\prime \prime }\left(\omega \right)=\epsilon^{\prime }\left(\omega \right)-j\frac{\sigma (\omega )}{\omega \epsilon_{0}}$, and is measured by the dielectric probe kit through measurement of $\epsilon^{\prime }\left(\omega \right)$ and $\epsilon^{\prime \prime }\left(\omega \right)$. The dielectric constant $\epsilon^{\prime }\left(\omega \right)$ is the real part of the complex permittivity. The effective conductivity σ(ω) can be easily calculated from the loss index $\epsilon^{\prime \prime }\left(\omega \right)$ for a given angular frequency ω, where $\epsilon_{0}$ is the dielectric constant of free space.

### 2.3. Microwave Experimental Method

**Figure 1:**
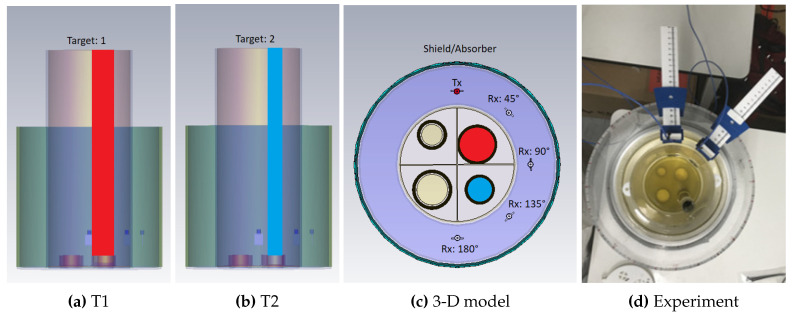
The modelled 3-D simulated and experimental configuration used to observe the transmitted signal differences caused by different dielectric targets: Drawings of (**a**) Target T1 (r = 15mm), (**b**) Target T2 (r = 10mm), and (**c**) the 3-D model used in simulations; (**d**) Photo of the experimental setup shown in these drawings.

The microwave experimental system to assess the impact of contrast agents is shown in Figure 1, and is comprised of two concentric cylindrical tanks with an inner and outer radius of 60 and 100 mm, respectively. The tank is made of acrylic, and the height of the inner and outer tanks are 300 and 200 mm, respectively. The base of the inner tank contains a target holder designed to support four liquid-filled targets at fixed locations. Two targets of the same height of 300mm but different radii are used in this study, Target 1 (T1) with radius r=15 mm, and Target 2 (T2) with r=10 mm. The targets are positioned in a custom acrylic cap which is attached on top of the inner tank at different locations. Outside the target cylinder, the inner tank is filled with safflower oil which has very low dielectric properties and corresponds to a homogeneous high adipose (fatty) tissue layer. The outer tank is filled with a 9/1 mixture of glycerol and RO water, in which the transmitting and receiving antenna are immersed. This mixture has a high loss tangent, and is chosen to minimise multipath signals [51]. To minimise multipath signals and external interference further, we used a microwave absorbing sheet (1mm thickness and height of 150 mm) around the periphery of the 200 mm tank and covered the external face with aluminium foil.

The system uses printed monopole antennas that operate in the frequency range between 1.0 and 3.0 GHz when immersed into the glycerol–water mixture [51]. The antennas are designed with a funnel shaped patch constructed on a FR-4 substrate and a partial ground. These antennas were designed to be small with a 180 mm^2^ surface area, which may help prevent multipath signals. Also, smaller antennas allow more elements in an antenna array, hence increasing the accuracy of received data. The SMA connectors were soldered on to the bottom face of the antennas and were joined to flexible coaxial cables with a dedicated internal O-ring, which prevented the coupling liquid leaking into the connectors. L-shaped calipers were used to position the antennas and move radially to take concentric scattering measurements. The experimental process used a fixed transmitter and a receiver which was radially positioned between 45${}^{\circ }$ and 180${}^{\circ }$ with respect to the tank centre. For each target scenario, we measured at four angles (45${}^{\circ }$, 90${}^{\circ }$, 135${}^{\circ }$, and 180${}^{\circ }$), allocating a time interval for the forward scattering data to settle before restarting the average, and repeated the process 5 times to assess the variability in our measurements. The antennas were connected to a two-port vector network analyser (VNA), and S-parameter ($S_{\theta }$) measurements were recorded between 1.0 and 3.0 GHz with 201 sample points. The intermediate frequency (IF) was 200 Hz and the average factor set to 10.

For every angle, the cylindrical target T1 (or smaller target T2) was filled with different materials prior to taking measurements at the next interval. These included samples of salt concentrations (10–50 mg/mL) dissolved in 60% glycerol water mixture, which were used as a reference in this study to investigate the correlation between dielectric contrast and transmitted signals difference. Furthermore, MWNT and MWNT-OH of diameter 20–30 nm and length 10–30 μm (Cheap Tubes, Grafton, VT, USA) at a concentration of 2 mg/mL dispersed in a 60% glycerol–water mixture were used as targets to correlate their contrast enhancement measured from dielectric spectroscopy with their impact on the signals recorded by the receiving antenna. The specific concentration was selected because of previous nanoparticle spectroscopy studies showing large dielectric contrast [32,35].

We also simulated these experiments in CST Microwave Studio, in order to study the impact of the contrast enhancement on transmitted signals in the absence of measurement errors. The simulated 2PMW setup was imported from STL files preserving the geometry of the experiment as shown in Figure 1. The phantom liquids with or without the contrast agents were assigned dielectric properties based on their dielectric spectroscopy measurements, while the acrylic used to model the imaging tank was assigned dielectric values taken from CSTs library of materials. The antennas were fully modelled as in [51]. Open space boundary conditions were used to terminate the computational domain outside the imaging tank. CSTs frequency solver was used with tetrahedral meshing, and with an initial mesh size of 10${}^{5}$ cells which evolved to 10${}^{6}$ cells after adaptive mesh refinement with a minimum and maximum of four and eight passes, respectively.

To quantify this impact, we plot (as function of frequency) the difference in transmitted signal for a given measurement angle, $\Delta S_{\theta }=\left|S_{\theta ,homog}-S_{\theta ,contrast}\right|$, where $S_{\theta ,homog}$ and $S_{\theta ,contrast}$ are the transmission coefficients at each receiver location in the absence (target filled with 60% glycerol–water mixture only) or presence of the contrast agent (target filled with the MWNT or salt solutions).

## 3. Results

### 3.1. Size Characterisation for MWNT and MWNT-OH NPs

**Figure 2:**
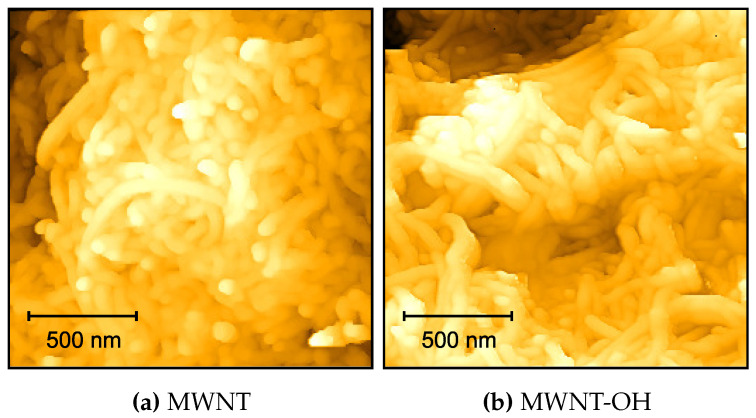
Atomic force microscopy (AFM) images of (**a**) MWNT and (**b**) MWNT-OH at a concentration of 2 mg/mL with scanned dimension of 1.7 μm × 1.7 μm.

AFM images are shown in Figure 2 and suggest that these nanomaterials have a tube/rod-like structure in nature. Moreover, analysis of the AFM micrographs gave an average diameter tube size of 32nm and 26nm for MWNT-OH and MWNT, respectively, which agrees with the width stated by the manufacturers (see Table 1). It was difficult to measure the length of CNTs because of their entanglement. However, the diameter of the CNTs obtained agreed with stated manufacturer size.

### 3.2. Dielectric Spectroscopy Measurements

Dielectric spectroscopy measurements with the probe kit were used to determine the level of dielectric contrast caused by the salt or MWNTs. As explained in the Methodology section, the dielectric properties were characterised between 1.0 and 4.0 GHz. A mixture consisting of 60% glycerol and 40% water was chosen as the background liquid in which the agents were dispersed, as our experiments have shown that this mixture improves the stability of colloidal dispersions. Furthermore, the dielectric properties of 60% glycerol–water mixture are similar to breast tumour tissue with a value of 56.6 at 1.0 GHz, which makes this choice relevant to a realistic scenario of detecting contrast agents in the tumour area.

**Figure 3:**
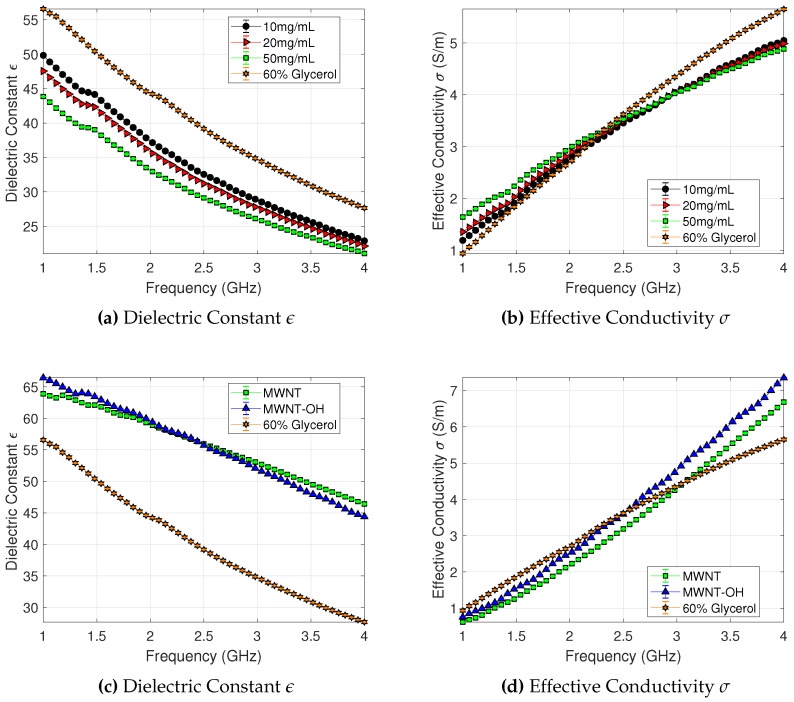
Microwave dielectric properties of the target materials used for dielectric contrast enhancement. (**a**) Dielectric constant, and (**b**) effective conductivity of 60% glycerol mixed with a range (10 mg/mL–50 mg/mL) of salt concentrations. (**c**) Dielectric constant, and (**d**) and effective conductivity of 60% glycerol suspensions with basic (MWNT) and functionalised (MWNT-OH) multi-walled carbon nanotubes at 2 mg/mL. The average values over 10 measurements (n = 10) of each salt solution and NP dispersion samples are plotted with ±1 $\sigma_{std}$.

Figure 3 plots the dielectric constant and conductivity vs. frequency for different concentrations of our contrast agents suspended/dissolved in 60% glycerol–water mixtures. As expected, the addition of salt decreases the dielectric constant over the whole frequency range, and this decrease is monotonic with respect to salt concentrations between 10–50 mg/mL. Conversely, the conductivity is increased for frequencies up to 2.0 GHz. At 1.0 GHz, for example, the dielectric constant drops from 56.4 for 60% glycerol to 44.8 for 60% glycerol with 50mg/mL dissolved salt. The effective conductivity increase is 20.1%, 39.7%, and 68.0% for 10, 20 and 50 mg/mL salt concentration, respectively. The increase in conductivity at lower frequencies is dependent on free drift ions while dielectric loss is prominent [62] at higher frequencies. In particular, the dielectric loss is masked at lower frequencies due to ionic conduction, but becomes more noticeable as frequency increases [62,63].

The dielectric values plotted in Figure 3 are averaged over 10 measurements for each salt solution sample. The measurement variations ($\sigma_{std}$) in all samples are small compared to the measured values. The standard deviation of the dielectric constant of the 60% glycerol mixture at 1.0 GHz is ±0.005. Homogeneous liquids tend to show small measurement variation due to isotropy of the material. The $\sigma_{std}$ of the dielectric constant at 1.0 GHz for saline samples are ±0.054, ±0.034, and ±0.027 for ascending concentrations of salt. The measurement variation increased with the addition of salt in these solutions and this level of error is similar throughout the frequency ranges, suggesting that the increase in salt (and thus in conductivity) may lead in an increase in the measurement uncertainty.

As discussed in the Introduction, CNTs have been previously proposed as contrast agents for microwave imaging, and their dielectric properties have been measured when dispersed in liquid or solid phantoms. However, there is no study that looks at the effect of functionalisation. MWNTs are functionalised to increase their water association, which leads to better dispersion and thus more biocompatibility. The dielectric properties of MWNTs dispersed in 60% glycerol at 2 mg/mL are presented in Figure 3c,d. Both samples show significant increase in the dielectric contrast with respect to the background 60% glycerol–water mixture over the measured frequency range, and the increase is more pronounced for higher frequencies (e.g., 12.93% and 17.41% for MWNT and MWNT-OH, respectively, at 1.0 GHz, and 67.79% and 60.47% at 4.0 GHz). Similar to the plots for salt concentrations, the dielectric values plotted in Figure 3 are averaged over 10 measurements of MWNT and MWNT-OH samples, and the measurement variations ($\sigma_{std}$) in all samples are small compared to the measured values. The $\sigma_{std}$ of the dielectric constant at 1.0 GHz for the MWNT and MWNT-OH NP samples are ±0.043 and ±0.092, respectively. At 4.0 GHz, the $\sigma_{std}$ of the dielectric constant for MWNT and MWNT-OH samples are ±0.044 and ±0.022, respectively.

The hydroxylated NPs show slightly larger contrast in comparison to the unfunctionalised multi-wall NPs up to 2.0 GHz, but this trend is reversed for higher frequencies. Dielectric properties are increased by functionalised MWNTs because dispersion properties of MWNTs are improved in 60% glycerol. At higher frequencies, however, it appears that more pristine CNTs lead to higher dielectric constant. The large contrast increase suggests that these NPs can impact transmitted and received signals in a microwave system.

### 3.3. Microwave System Measurements

As detailed in the Methodology section, microwave measurements were taken with our 2PMW setup, where the transmitter was at a fixed position and the receiver was positioned at angles 45${}^{\circ }$–180${}^{\circ }$ relative to the transmitter. Different-diameter cylinders T1 and T2 (see Figure 1) were used as targets to understand how the volumetric difference affected the system response. The 60% glycerol–water mixture was used as the background, “non-agent” case.

#### 3.3.1. Salt (NaCl)

**Figure 4:**
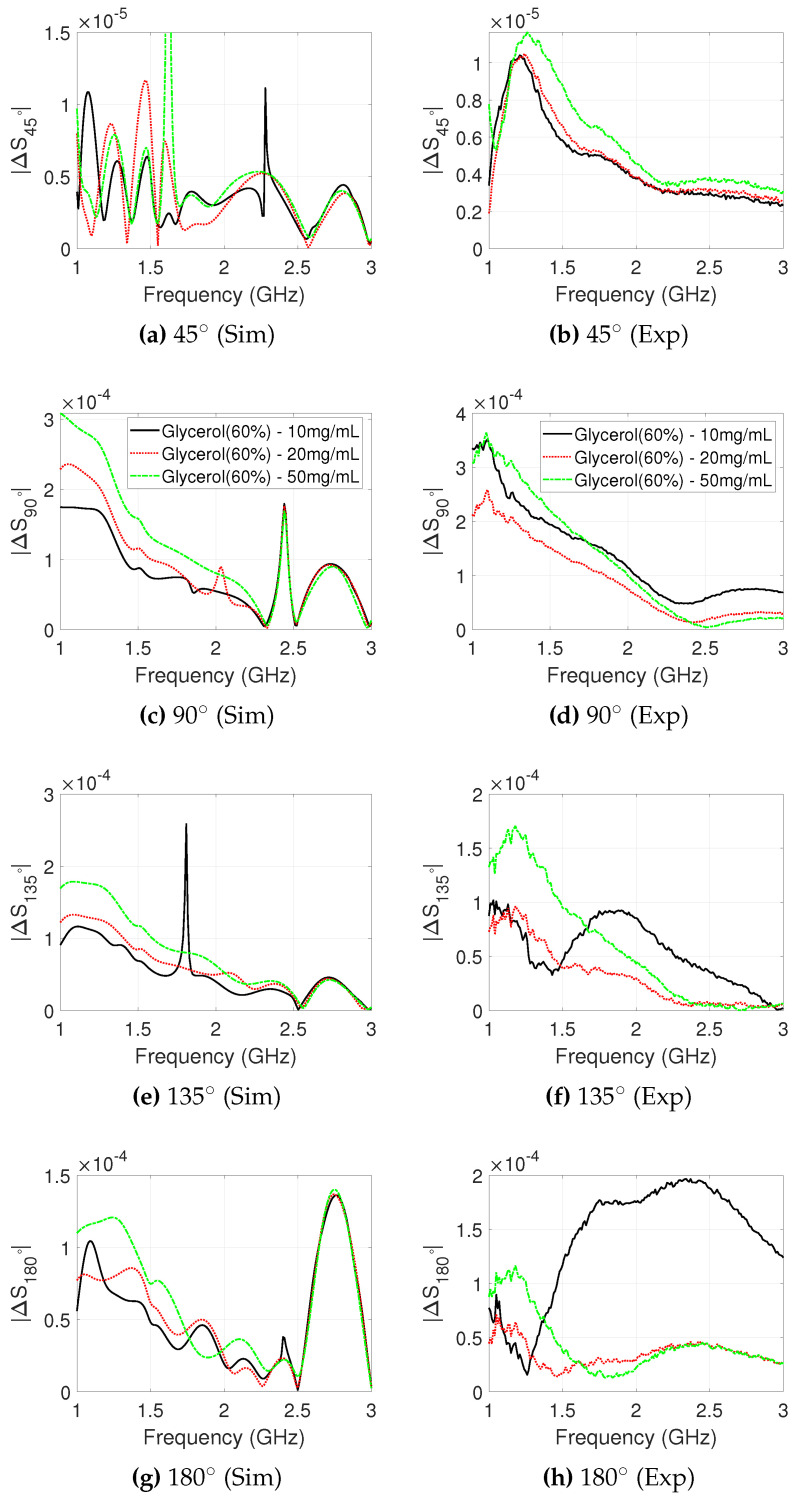
Difference in received signal strength between “no-target” and “target T1” cases, plotted for each receiver position against frequency. T1 is filled with salt concentrations between 10 and 50mg/mL dissolved in 60% glycerol. The simulated (**left**) and experimental (**right**) signal differences are plotted between 1.0 and 3.0 GHz, which is the operational frequency range for the employed antennas.

Salt concentrations between 10 and 50mg/mL dissolved in 60% glycerol were used inside either the T1 or T2 target cylinders to correlate their dielectric contrast effect with the impact on microwave signals recorded by the 2PMW system. The difference between the background (60% glycerol) and the inclusion (60% glycerol and salt concentration) was calculated at each receiver location. Figure 4 shows the difference in received signal strength at each receiver position due to the different salt concentrations, for both experimental and simulated data.

These results confirm that larger concentrations of salt cause stronger signal differences at the lower end of the frequency spectrum, where the antenna operates optimally and signal losses are lower. Impact on higher frequencies is difficult to detect with these experiments, due to the very low signal levels. For example, the experimental signal difference at 45${}^{\circ }$ shows a maximum value of −98.4 dB, which is close to the noise level of the VNA. For the more accurate lower frequency range of up to 2 GHz, receiver angles 90${}^{\circ }$–180${}^{\circ }$ show that the signal difference increases as the salt concentration increases, particularly for the highest concentration of 50 mg/mL. The level of experimentally measured signal difference agrees well with the simulation results, although the two signals are very different. There are however cases of fairly good agreement between the measured and simulated signals, such as in the range 1.2–1.5 GHz for the 135${}^{\circ }$ location.

**Figure 5:**
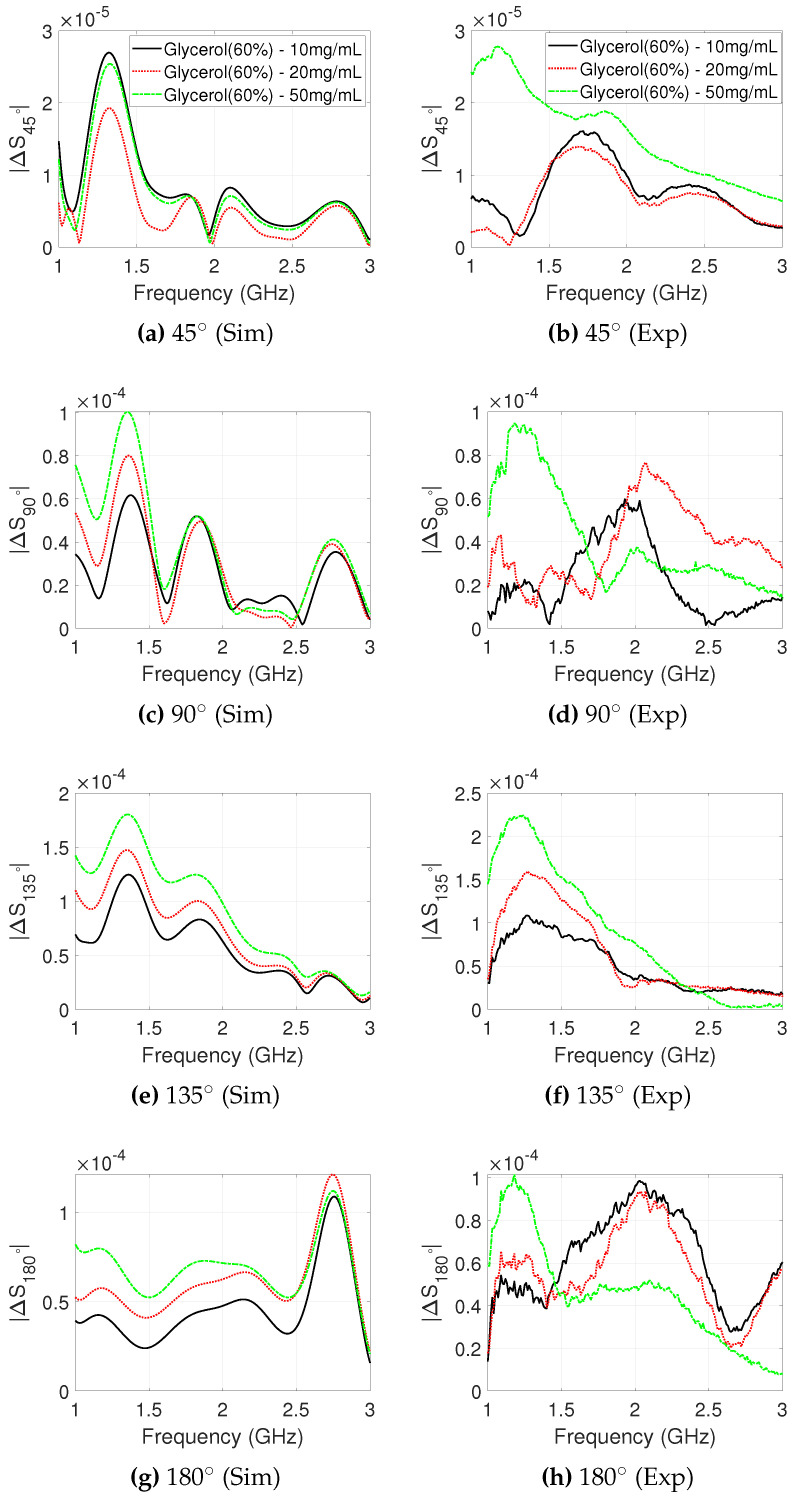
Difference in received signal strength between “no-target” and “target T2” cases, plotted for each receiver position against frequency. T2 is filled with salt concentrations between 10–50 mg/mL dissolved in 60% glycerol. The simulated (**left**) and experimental (**right**) signal differences are plotted between 1.0–3.0 GHz, which is the operational frequency range for the employed antennas.

The received signal difference using the smaller target cylinder, T2, is plotted in Figure 5 for the different receiver positions. In this case, $\Delta S_{45^{\circ }}$ (Figure 5a) is less than −90.0 dB. The signal difference at position 90${}^{\circ }$ peaks around −80.0dB for the 50mg/mL salt target, for both experimental and simulation data. Simulated differences at position 90${}^{\circ }$ do show a clear monotonic behaviour between 1.0 and 1.5 GHz, but this is less evident in the experimental results, and particularly for the two lower NaCl concentrations. The 10mg/mL experimental difference at position 90${}^{\circ }$ is unexpected for both T1 and T2 targets. This is in contrast to the simulated difference at 90${}^{\circ }$, where again a monotonic behaviour is observed.

Significant signal differences for both experimental and simulated signals are observed for $\Delta S_{135^{\circ }}$, with a clear monotonic dependence on salt concentrations. The peak experimental values $S_{135^{\circ }}$ are −79.2 dB, −75.9 dB, and −73.2 dB for 10 mg/mL, 20 mg/mL, and 50 mg/mL salt concentrations, respectively, at 1.3 GHz. Experimental signal differences $\Delta S_{180^{\circ }}$ are concentration-dependent between 1.0 and 1.4GHz, with a maximum contrast of −80.0 dB for the 50 mg/mL salt target.

#### 3.3.2. MWNT and MWNT-OH Suspensions

The signal differences due to the salt concentrations analysed in the previous section showed that a measured dielectric contrast decrease in the range of 12–26% can have a detectable impact on received signal strength measured by the 2PMW system. We now repeat this analysis to observe the effect of the MWNTs suspensions in the 60% glycerol background for targets T1 and T2.

**Figure 6:**
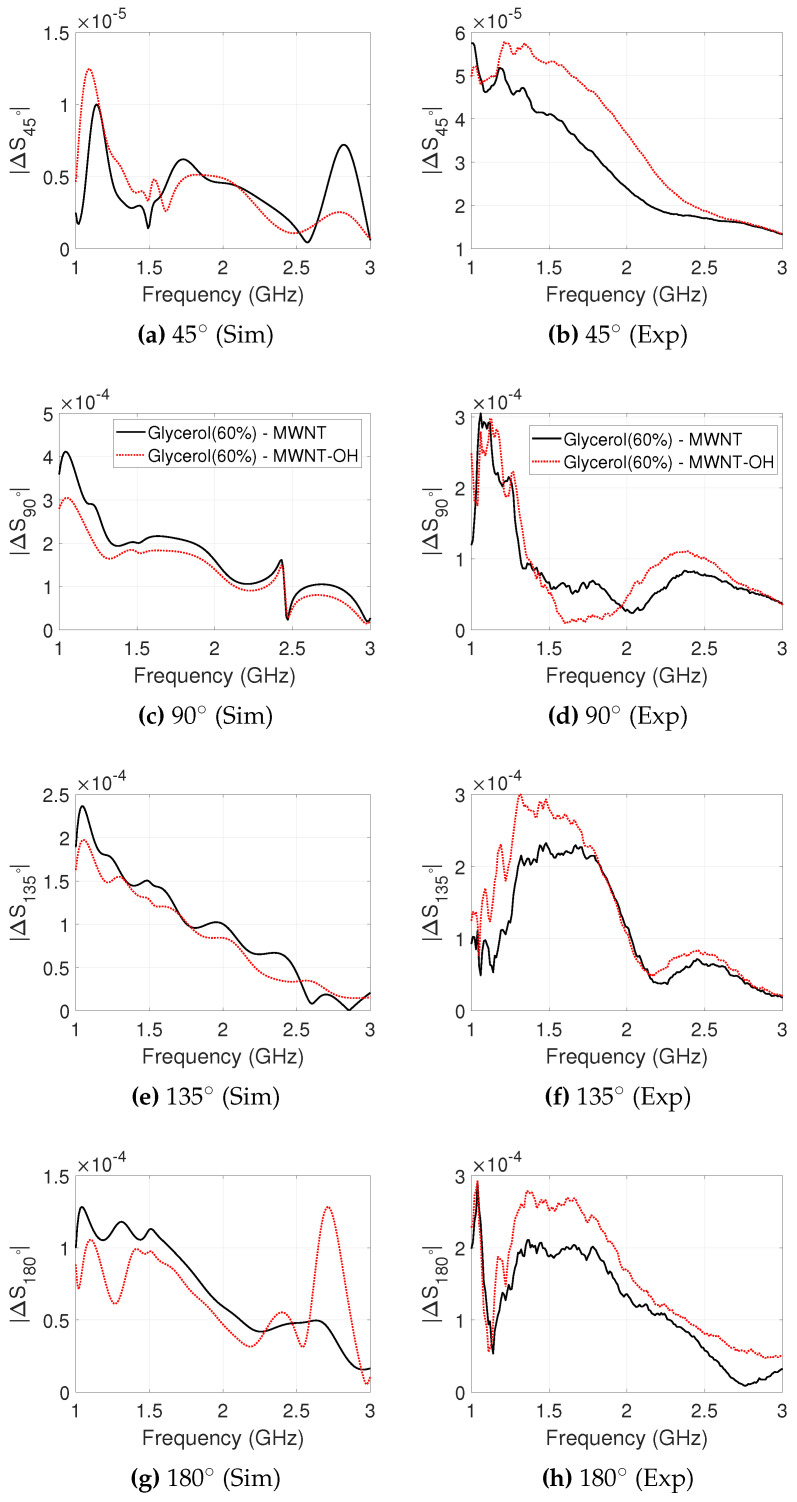
Difference in received signal strength between “no-target” and “target T1” cases, plotted for each receiver position against frequency. T1 is filled with MWNT and MWNT-OH colloidal suspensions with a concentration of 2 mg/mL dispersed in 60% glycerol. The simulated (**left**) and experimental (**right**) signal differences are plotted between 1.0–3.0 GHz, which is the operational frequency range for the employed antennas.

Simulated and experimental signal differences for MWNT and MWNT-OH dispersions in target T1 are plotted in Figure 6. Similar to the cases with salt, the receiver position at 45${}^{\circ }$ is affected the least by the contrast, with experimental and simulated signal differences showing a maximum contrast of −84.4 dB and −97.1 dB, respectively. The simulated $\Delta S_{90^{\circ }}$ has a maximum value of −67.8 dB (MWNT), while the maximum experimental $\Delta S_{90^{\circ }}$ is −70.5 dB. The signals of $\Delta S_{90^{\circ }}$ shows good agreement between simulation and experiment in 1.0–1.5 GHz, although the simulated MWNT-OH target response is lower in magnitude. MWNT and MWNT-OH suspensions show a maximum contrast of −68.0 dB and −70.5 dB for position 135${}^{\circ }$, respectively, and good agreement is observed for $\Delta S_{135^{\circ }}$ between simulation and experiment in the range 1.5–2.0 GHz, but not between 1.0 and 1.5 GHz. Experimental and simulated $\Delta S_{180^{\circ }}$ show good agreement, although the peak value is larger for the experiment.

**Figure 7:**
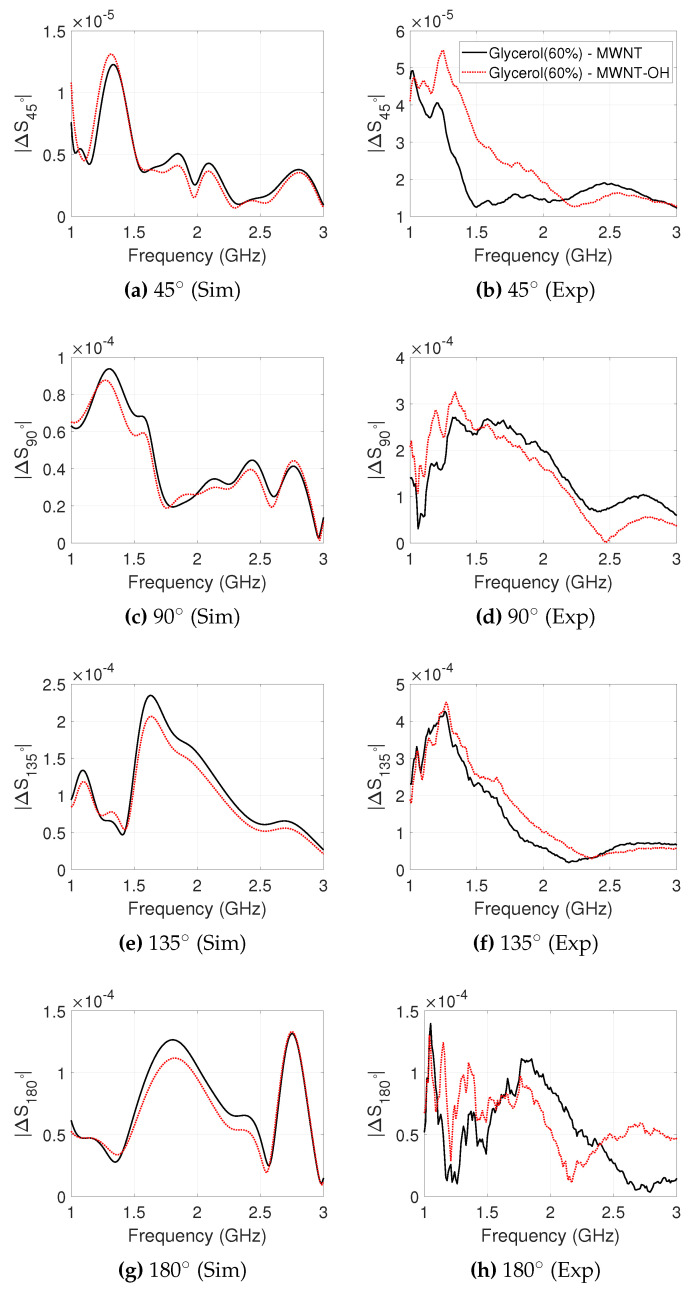
Difference in received signal strength between “no-target” and “target T2” cases, plotted for each receiver position against frequency. T2 is filled with MWNT and MWNT-OH colloidal suspensions with a concentration of 2 mg/mL dispersed in 60% glycerol. The simulated (**left**) and experimental (**right**) signal differences are plotted between 1.0 and 3.0 GHz, which is the operational frequency range for the employed antennas.

The simulated and experimental signal differences for MWNT and MWNT-OH suspension in target T2 are plotted in Figure 7. The simulated $\Delta S_{90^{\circ }}$ shows a maximum contrast of −80.5 dB, however the experimental $\Delta S_{90^{\circ }}$ shows a significantly larger signal difference of −69.6 dB. The experimental $\Delta S_{135^{\circ }}$ shows a large difference due to the MWNT suspension, with a maximum of −67.1dB and −67.5 dB for MWNT and MWNT-OH suspensions, respectively. In simulation, the maximum difference at position $135^{\circ }$ for MWNT and MWNT-OH targets are −72.0 dB and −73.7 dB, respectively. The simulated difference is lower in magnitude and the peak is shifted in frequency in comparison with the experimental signal difference. The experimental and simulated transmission contrast observed at $\Delta S_{180^{\circ }}$ have some agreement between 1.2 and 2.5 GHz, however the experimental difference has large variation between frequency points.

## 4. Discussion

### 4.1. Dielectric Spectroscopy Analysis

The contrast in conductivity due to the salt or the MWNTs is surprisingly lower than when these are in water. Glycerol forms hydrogen bond networks when mixed in water, which has an unusual increase in dielectric loss at higher frequencies, leading to a departure from Debye-type relaxations [64]. This phenomenon is called excess wing and is caused by glass forming materials. Depending on the glycerol–water mixture ratio, interactions between glycerol–glycerol regions, water–water regions and glycerol–water regions affect the dielectric relaxation [65]. This causes two overlapping dielectric relaxations process; alpha(α) relaxation, which is caused by the glycerol–water and glycerol–glycerol interactions, and beta(β)relaxations, which is caused by water–water interactions [66].

MWNTs are generally conductive because of delocalised electrons. These electrons can travel a distance without being scattered, a phenomenon known as ballistic conduction. MWNTs are shown to be ballistic conductors at room temperatures, with mean free paths of the order of tens of microns [67]. Longer and thicker CNTs ensure a better conduction pathway, hence larger dielectric properties are observed with MWNTs. Also, there is an increase in space charge polarisation, due to the accumulation of electrons at the surface, which has an additive effect [68].

### 4.2. Microwave System Analysis

We have built the 2PMW system as a simple tool to assess dielectric constant of phantoms with and without contrast agents. Our approach relies on observations of the raw scattering data from the 2PMW system, rather than attempting to reconstruct images from the system. Reconstructions with limited view data are susceptible to various measurement and imaging model errors and would require a more sophisticated system. In contrast, our approach can offer a simple way to assess the NPs impact directly on signals similar to what a MWI system would record, rather than relying on dielectric spectroscopy measurements which are not directly correlated with MWI measurements. Interpreting these experimental signal differences is also challenging, however, as they are caused by various interactions of the transmitted signal with the system and target. The opportunity to observe contrast is between 1.0 and 1.5 GHz, as our 2PMW system’s antennas are better matched in this band [51] and the 60% glycerol–water mixture becomes very lossy for higher frequencies (its loss tangent tanδ is in the range 0.4–0.8 for the 1.5–3.0 GHz band).

As shown in Figure 1, the experimental setup was built to assess the impact of contrast enhancement for two different targets and locations. As the two cases differ both is size and location, they cannot be compared directly. These two cases represent only two out of many possible responses from the contrast-enhanced target region. The impact of the target’s size and location on the contrast-enhanced signals is of course a very important topic which we intend to examine in a future study. This will include more Tx/Rx pairs and will aim at estimating the target size and position using our MWI algorithms. We also note that the dielectric spectroscopy results show enhanced contrast at higher frequencies than the range of the 2PMW. According to our dielectric spectroscopy measurements, MWNTs induce maximum dielectric contrast in high frequencies, where high tissue losses in a realistic medical MWI system may result in received signals below the noise level of the VNA. Our 2PMW experiments allow assessing the impact of contrast enhancement in frequencies that could be exploited by a practical MWI data acquisition system.

Although simulations do not agree well with experiments due to the very low signal levels, the observed agreement in order of magnitude and frequency dependence trend suggests that the system can provide an estimation of the impact of the contrast agent enhancement on the microwave signals. Simulation results exhibit signal peaks at certain frequencies which can only be interpreted as numerical errors. They are mostly present for the lowest contrast enhancement case and at high frequencies, where the lowest signal differences are produced. In addition to calculating very low signals, the simulations model the materials as frequency dispersive based on their dielectric probe measurements, and numerical modelling of dispersive materials is more susceptible to numerical error.

As the microwave signals of interest are quite weak and thuThis averaging was performed to account for random experimental errors such as those from connectors and cables. To minimise systematic errors from the measurement due to the VNA, the cables and the connectors, we calibrated our measurement up to the plane of the antennas using a standard calibration procedure with Keysight’s E-Cal module, without assessing further the accuracy of this calibration process. Overall, we have found that variations between measurements were insignificant compared to the level of signals due to the targets. For example, between angles 135${}^{\circ }$ and 180${}^{\circ }$ where we identify signal contrast, the measurement variations at 1.0 GHz were below −88 dB for all salt and NP target samples and both T1 and T2 targets. Furthermore, measurement variations for all salt targets at 1.0 GHz and all 10–50 mg/mL concentrations in T1 and T2 were below −100 dB. The lower measurement variations for salt targets relative to NP targets is expected as the salt mixtures are more homogeneous than the NP dispersions.

It is also important to note that the experiments were performed in a way to ensure that the contrast medium was injected and removed inside the cylindrical tubes without affecting the setup or the antennas, so that these signal differences are only due to the contrast enhancement. The dielectric spectroscopy data of MWNT and MWNT-OH suspensions suggests that they can produce detectable signal differences, which are indeed observed in our simulation and experimental results, with small differences between MWNT and MWNT-OH. The likely aggregation of these MWNT may have contributed to a non-homogenous dispersion leading to mismatch between simulation and experiment, besides inevitable experimental measurement errors. As an example, there is much better agreement between simulation and experiment for salt than for MWNT, as observed for $\Delta S_{135^{\circ }}$ and both targets T1 and T2.

### 4.3. Contributions

This study presented two experimental approaches to test the efficacy of candidate contrast agents for microwave sensing and imaging. First, the selected contrast targets were dispersed inside a glycerol/water mixture with dielectric properties resembling malignant tissue, and the properties of the resulting solutions were measured with dielectric spectroscopy. These measurements were also used to account for the agents’ properties in CST simulations of a microwave experimental system, which was proposed to study the impact of the contrast agents on the received signals. Simulations and experimental measurements were conducted with different target sizes and locations to observe the effect of contrast enhancement for salt and MWNTs, and correlate it to the dielectric measurements.

Our results show that a dielectric contrast of 12–26% induced by the salt concentrations results in signal difference above −70 dB. MWNTs induce significant dielectric contrast, e.g., around 13–17% at 1.0GHz, which again led to detectable signal difference above −75 dB. Although the dielectric contrast for the MWNT suspensions increases for higher frequencies, the signal difference strength is mostly observed in the range 1.0–2.0 GHz. This is not only due to the greater signal losses in higher frequencies, but also because the antennas used by the microwave system operate best in this range. These results suggest that achieving differences above 10% in dielectric contrast as measured by the probe can be exploited by a MWI system. Notably our 2PMW system is able to detect the contrast of isotropic salt solutions and, more importantly, the contrast of non-homogenous MWNT/MWNT-OH colloidal dispersions, which is a good indicator that contrast enhancement has significant potential for microwave imaging and sensing techniques. This important finding can guide future work on what contrast levels should be sought in dielectric spectroscopy measurements used to assess NPs as contrast agents.

Based on the above, the 2PMW system is a useful tool in reliably screening nanomaterials for microwave contrast in a robust way, to identify optimal variation, concentration and frequency for maximum contrast enhancement. It offers the possibility to screen candidate nanomaterials as contrast agents and develop them to demonstrate high dielectric constant while maintaining their dispersion in phantoms. Nanomaterial dispersion in aqueous or oil-based solutions may result in non-homogenous mixtures, which imposes limitations for the coaxial probe method that requires homogenous samples. For example, we have observed in our experiments that MWNTs tend to aggregate inside the 60% glycerol mixture in which they are added, and this results in a non-linear dependence of the dielectric contrast enhancement on the NPs concentration. Potential NPs for contrast-enhanced cancer detection, therefore, will require optimisation for size, functionalisation for blood compatibility, as well as addition of moieties for receptor targeting. All these processes will require assessing their impact on dielectric properties, which can be conducted with the proposed approach.

## Figures and Tables

**Table 1:** The manufacturer-stated and AFM-calculated width of basic and functionalised multi-walled carbon nanotubes (MWNT and MWNT-OH, respectively).

CNT	Stated Width (nm)	AFM Width (nm)
MWNT	20 – 30	26.4 ± 5.1
MWNT-OH	20 – 30	32.1 ± 4.7
